# Preventive Service Usage and New Chronic Disease Diagnoses: Using PCORnet Data to Identify Emerging Trends, United States, 2018–2022

**DOI:** 10.5888/pcd21.230415

**Published:** 2024-07-03

**Authors:** Sandra L. Jackson, Akaki Lekiachvili, Jason P. Block, Thomas B. Richards, Kshema Nagavedu, Christine C. Draper, Alain K. Koyama, Lindsay S. Womack, Thomas W. Carton, Kenneth H. Mayer, Sonja A. Rasmussen, William E. Trick, Elizabeth A. Chrischilles, Mark G. Weiner, Pradeep S. B. Podila, Tegan K. Boehmer, Jennifer L. Wiltz, on behalf of PCORnet Network Partners

**Affiliations:** 1 Division for Heart Disease and Stroke Prevention, National Center for Chronic Disease Prevention and Health Promotion, Centers for Disease Control and Prevention, Atlanta, Georgia; 2 National Center for Chronic Disease Prevention and Health Promotion, Centers for Disease Control and Prevention, Atlanta, Georgia; 3 Division of Chronic Disease Research Across the Lifecourse, Department of Population Medicine, Harvard Pilgrim Health Care Institute, Harvard Medical School, Boston, Massachusetts; 4 Division of Cancer Prevention and Control, National Center for Chronic Disease Prevention and Health Promotion, Centers for Disease Control and Prevention, Atlanta, Georgia; 5 Division of Therapeutics Research and Infectious Disease Epidemiology, Department of Population Medicine, Harvard Pilgrim Health Care Institute, Harvard Medical School, Boston, Massachusetts; 6 Division of Diabetes Translation, National Center for Chronic Disease Prevention and Health Promotion, Centers for Disease Control and Prevention, Atlanta, Georgia; 7 Division of Reproductive Health, National Center for Chronic Disease Prevention and Health Promotion, Centers for Disease Control and Prevention, Atlanta, Georgia; 8 Louisiana Public Health Institute, New Orleans; 9 The Fenway Institute, Fenway Health and the Division of Infectious Diseases, Department of Medicine, Beth Israel Deaconess Medical Center/Harvard Medical School, Boston, Massachusetts; 10 Johns Hopkins School of Medicine, Baltimore, Maryland; 11 Center for Health Equity and Innovation, Cook County Health, Chicago, Illinois; 12 Department of Epidemiology, College of Public Health, University of Iowa, Iowa City; 13 Department of Population Health Sciences, Weill Cornell Medicine, New York, New York; 14 Office of Informatics and Information Resource Management, National Center for Chronic Disease Prevention and Health Promotion, Centers for Disease Control and Prevention, Atlanta, Georgia; 15 Office of Public Health Data, Surveillance, and Technology, Centers for Disease Control and Prevention, Atlanta, Georgia; 16 US Public Health Service, Atlanta, Georgia

## Abstract

**Background:**

Data modernization efforts to strengthen surveillance capacity could help assess trends in use of preventive services and diagnoses of new chronic disease during the COVID-19 pandemic, which broadly disrupted health care access.

**Methods:**

This cross-sectional study examined electronic health record data from US adults aged 21 to 79 years in a large national research network (PCORnet), to describe use of 8 preventive health services (N = 30,783,825 patients) and new diagnoses of 9 chronic diseases (N = 31,588,222 patients) during 2018 through 2022. Joinpoint regression assessed significant trends, and health debt was calculated comparing 2020 through 2022 volume to prepandemic (2018 and 2019) levels.

**Results:**

From 2018 to 2022, use of some preventive services increased (hemoglobin A_1c_ and lung computed tomography, both *P* < .05), others remained consistent (lipid testing, wellness visits, mammograms, Papanicolaou tests or human papillomavirus tests, stool-based screening), and colonoscopies or sigmoidoscopies declined (*P* < .01). Annual new chronic disease diagnoses were mostly stable (6% hypertension; 4% to 5% cholesterol; 4% diabetes; 1% colonic adenoma; 0.1% colorectal cancer; among women, 0.5% breast cancer), although some declined (lung cancer, cervical intraepithelial neoplasia or carcinoma in situ, cervical cancer, all *P* < .05). The pandemic resulted in health debt, because use of most preventive services and new diagnoses of chronic disease were less than expected during 2020; these partially rebounded in subsequent years. Colorectal screening and colonic adenoma detection by age group aligned with screening recommendation age changes during this period.

**Conclusion:**

Among over 30 million patients receiving care during 2018 through 2022, use of preventive services and new diagnoses of chronic disease declined in 2020 and then rebounded, with some remaining health debt. These data highlight opportunities to augment traditional surveillance with EHR-based data.

**Summary**

**What is already known on this topic?**

Preventive services and screenings facilitate prevention, early detection, and treatment of chronic diseases, but the COVID-19 pandemic disrupted care.

**What is added by this report?**

This study of over 30 million US adults during 2018 through 2022 leveraged electronic health record (EHR) data from PCORnet, a national network, to examine use of preventive services and diagnoses of new chronic disease, including trends over time and stratifications by demographic characteristics. Preventive services and chronic disease diagnoses declined during 2020 and subsequently rebounded to nearly prepandemic levels but lagged behind prepandemic levels for some services and diagnoses.

**What are the implications for public health practice?**

EHR data can augment traditional surveillance and highlight key emerging patterns in health care service usage and in diagnosis of chronic disease. Such data may help optimize prevention and outcomes.

## Introduction

Chronic diseases are primary causes of illness, disability, and death in the United States, and prevention or early detection can facilitate treatment and improve outcomes (1). The US Preventive Services Task Force (USPSTF) recommends screening for chronic diseases including hypertension, diabetes, breast cancer, cervical cancer, colorectal cancer, and lung cancer (2). However, the COVID-19 pandemic disrupted health care access and impaired the ability of US adults to obtain preventive care such as screening for chronic disease, resulting in health debt (ie, missed services, screenings, treatment, or diagnoses) that could have long-lasting negative health consequences (1,3–5). Given health care system burden, as well as concerns about exposure to COVID-19 for at-risk populations during office visits, some experts recommended deferring screening during the most acute phase of the pandemic (6,7). The extent of the disruption, its duration, and its potential impact on subsequent chronic disease diagnoses have been difficult to assess by using existing public health data.

As a component of data modernization efforts, electronic health record (EHR) data have the potential to augment traditional chronic disease surveillance and provide timely and actionable data for public health policies and programs (8). In contrast to some existing surveys such as the National Health Interview Survey and the Behavioral Risk Factor Surveillance System (BRFSS), EHR data offer objective measurements of chronic disease status and screening practices (rather than self-report), large population sizes that allow for examination of rare events, and more timely insights. PCORnet, the National Patient-Centered Clinical Research Network, is an EHR-based network designed to generate high-quality, actionable evidence to support clinical care and high-priority research (9,10). As of 2020, PCORnet collected data from over 300 hospitals, 3,500 primary care practices, 1,000 community clinics, and 80 million patients, with broad geographic coverage across the US (9). Because of the large, diverse population receiving care in its participating health systems, PCORnet has the potential to complement traditional surveillance efforts.

The primary objectives of this study were to examine recent trends in use of preventive services and in new diagnoses of chronic disease and quantify health debt during the COVID-19 pandemic by using a large multisite cohort of US adults. A secondary objective was to explore and describe the usefulness of PCORnet data for chronic disease surveillance.

## Methods

### Participants and procedures

PCORnet is a distributed research network wherein participating health systems perform quarterly data updates and quality checks, and map raw clinical data to a standardized Common Data Model, enabling cross-site interoperability. With this standardization, modular statistical programs can query data across sites in a distributed model and combine results to produce aggregate reports. This cross-sectional study used a modular program (https://github.com/PCORnet-DRN-OC/Query-Details/) to generate site-level aggregate data in 2 separate queries; all results were returned to a coordinating center and combined. The first query examined preventive services (distributed in March 2023; N = 30,783,825 patients) and the second query examined new chronic disease diagnoses (distributed in June 2023; N = 31,588,222 patients). Both queries were performed by 36 PCORnet sites (each representing 1 or more health systems); a data refresh occurred between the 2 queries, resulting in additional patients (9,10). Adults aged 21 to 79 years who had at least 1 encounter at a PCORnet site from January 1, 2018, through December 31, 2022, were included (Supplemental Table 1, available at https://stacks.cdc.gov/view/cdc/156743). Diagnoses and services were identified by procedure codes (Current Procedural Terminology; Healthcare Common Procedure Coding Systems [CPT/HCPCS], 9th and 10th Revisions), diagnostic codes (International Classification of Diseases, 9th and 10th Revisions, Clinical Modification [ICD-9-CM and ICD-10-CM]), laboratory codes (Logical Observation Identifiers Names and Codes [LOINC]), or prescribing codes (National Drug Code [NDC]; vocabulary for normalizing prescription and over-the-counter drugs [RxNorm]). All codes are available at the GitHub link above.

### Measures

#### Preventive services

We examined 8 preventive services: 1) wellness visits; 2) glycated hemoglobin (HbA_1c_) laboratory tests; 3) lipid panel laboratory tests; 4) mammograms (among women); 5) Papanicolaou (Pap) tests or human papillomavirus (HPV) tests (among women); 6) low dose computed tomography (CT) scans for lung cancer; 7) colonoscopies or sigmoidoscopies; and 8) stool-based colorectal cancer screening tests, including fecal immunochemical tests or stool DNA-fecal immunochemical tests (eg, Cologuard). Results for mammograms and Pap tests/HPV tests are presented as percentages of all unique female patients; the other percentages are of all unique patients.

#### New chronic disease diagnoses

We examined new diagnoses of 9 chronic conditions — among patients with no prior record in PCORnet of each condition at any point since January 1, 2009. For new conditions in each year, exclusions were made for any period before that specific year. The 9 conditions were defined by using ICD codes unless specified: 1) type 2 diabetes mellitus (defined as HbA_1c_ value ≥6.5%, or an RxNorm or NDC code for a diabetes medication [other than metformin or a SGLT2 receptor antagonist, which are often used for conditions other than diabetes], or an ICD code for diabetes); 2) hypertension; 3) hypercholesterolemia (defined as starting a cholesterol medication by using RxNorm or NDC code); 4) breast cancer (among women); 5) cervical cancer (among women); 6) cervical intraepithelial neoplasia or carcinoma in situ (CIN/CIS) (among women); 7) lung cancer; 8) colorectal cancer; and 9) colonic adenoma. Some of these new diagnoses were merely new diagnoses in the PCORnet system, as some patients may have been previously diagnosed in a nonparticipating health system.

#### Health debt

Health debt was estimated by averaging the prepandemic (2018 and 2019) annual percentages of unique patients receiving each preventive service or new chronic disease diagnosis. Next, these prepandemic percentages were multiplied by the annual total unique patients per year (2020, 2021, and 2022) to calculate expected annual services or chronic disease diagnoses during the pandemic period if prepandemic levels of preventive services and diagnoses had continued. The observed annual numbers were then divided by the expected annual numbers, for each year individually (2020, 2021, and 2022), as well as collectively (the sum of observed services or diagnoses for 2020 through 2022 divided by the sum of expected services or diagnoses for 2020 through 2022). The resulting percentages indicate health debt if less than 100% (ie, observed services or diagnoses were less than expected).

#### Sociodemographic and clinical characteristics

Variables included age, sex (male, female, other/missing), and race and ethnicity (non-Hispanic American Indian or Alaska Native, non-Hispanic Asian, non-Hispanic Black, non-Hispanic multiple race, non-Hispanic Native Hawaiian or Other Pacific Islander, non-Hispanic White, other, missing, and Hispanic). Some US Census measures were linked at the ZIP Code Tabulation Area level, including urbanicity (isolated, small rural, large rural, urban, missing) based on Rural-Urban Commuting Area codes (https://depts.washington.edu/uwruca/) and socioeconomic status measured by Area Deprivation Index (ADI) (Quartile 1, ADI 0–38; Quartile 2, ADI 39–43; Quartile 3, ADI 44–49; Quartile 4, ADI 50–100; missing) (11,12). Clinical characteristics included body mass index (BMI, with obesity defined as BMI ≥30 calculated as weight in kilograms divided by the square of height in meters) and comorbidity status (by using up to 5-year diagnostic history for heart disease, diabetes, cancer, hypertension, mental health disorders, and smoking, for those with available data).

### Statistical analysis

Each PCORnet site executed the SAS-based modular statistical program query (SAS Institute Inc) on patient-level data that remained behind each health system’s firewall. The coordinating center combined all data across sites, and descriptive analyses were performed on the aggregate data in 2023 using Excel (Microsoft) and R (The R Foundation). Joinpoint regression was conducted to assess significant trends from 2018 to 2022 by using the National Cancer Institute’s Joinpoint Trend Analysis Software (version 5.0.2). Results were calculated overall and were stratified by select demographic characteristics. For example, results related to colorectal cancer screening and diagnoses were examined by age group to examine potential changes in relation to the 2021 USPSTF recommendation that lowered the colorectal cancer screening threshold from age 50 to age 45 (2). This activity was reviewed by the Harvard Pilgrim Health Care Institutional Review Board and by the Centers for Disease Control and Prevention (CDC), was deemed not human subjects research, and was conducted consistent with applicable federal law and CDC policy. The data for this study were shared by using existing data use agreements between sites involved. For data requests from outside collaborators, PCORnet has a standard process called the Front Door (https://pcornet.org/front-door/).

## Results

During 2018 through 2022, approximately 12 to 16 million adult patients per year received care in participating PCORnet sites (Table 1, preventive services query results; Supplemental Table 2, available at https://stacks.cdc.gov/view/cdc/156743, shows results for new chronic disease diagnoses). Of these, 59% were women and most (>75%) lived in urban areas. Over half were non-Hispanic White, approximately 16% were non-Hispanic Black, 13% were Hispanic, and 3% were non-Hispanic Asian (6%–9% missing, 2%–3% other; there was some variation by year). Among those with available BMI (~70%), approximately 40% had obesity.

**Table 1:** Demographic and Clinical Characteristics of Adult Patients in the Preventive Services Query, 36 National Patient-Centered Clinical Research Network (PCORnet) sites^a^, 2018–2022

Characteristic	2018	2019	2020	2021	2022
**All unique patients, n**	12,236,819	13,026,666	13,353,937	15,590,610	13,826,891
**Age, y^b^, %**					
21–34	24	24	25	24	24
35–39	9	9	9	9	9
40–44	8	8	8	8	8
45–49	9	8	8	8	8
50–64	30	29	29	28	28
65–79	21	21	21	22	23
**Sex^c^, %**					
Female	59	59	59	59	59
Male	41	41	41	41	41
**Race and ethnicity^d^, % **					
Non-Hispanic Asian	3	3	3	3	3
Non-Hispanic Black	16	16	15	15	16
Non-Hispanic White	59	59	58	56	57
Hispanic	13	13	13	13	13
**Body mass index^e^, %**					
Underweight (<18.5)	2	2	2	2	2
Normal weight (18.5–<25)	27	26	26	25	25
Overweight (25–<30)	32	31	31	31	31
Obese (≥30)	40	41	42	42	43
**Comorbidity (5-year history), %**					
Heart disease	11	11	11	10	12
Diabetes	10	11	11	10	12
Cancer	6	6	6	5	6
Hypertension	24	24	24	23	25
Mental health disorders	11	11	12	11	13
Smoking	13	13	13	12	13
**Urbanicity^c^, %**					
Isolated	2	2	2	2	2
Small rural	2	2	2	2	2
Large rural	6	6	6	6	6
Urban	84	83	82	80	76
**Socioeconomic status: Area Deprivation Index quartiles^c^, %**					
Quartile 1: 0–38	27	26	26	26	24
Quartile 2: 39–43	23	23	23	22	21
Quartile 3: 44–49	22	22	22	21	21
Quartile 4: 50–100	23	22	21	20	20

a National Patient-Centered Clinical Research Network (PCORnet) sites: Duke University, Medical University of South Carolina, Vanderbilt University Medical Center, Wake Forest Baptist Health, Allina Health, Medical College of Wisconsin, University of Iowa Healthcare, University of Kansas, University of Missouri HC, University of Nebraska, University of Texas SW Medical Center, University of Utah, University Medical Center New Orleans, Ochsner Health System, Children’s Hospital Colorado, Children’s Hospital of Philadelphia, Cincinnati Children’s Hospital, Nationwide Children’s Hospital, Nemours Children’s Hospital, Seattle Children’s Hospital, Lurie Children’s Hospital, Columbia, Cook County, Northwestern University, Fenway Health, Health Choice Network, OCHIN, Inc, Johns Hopkins University, Penn State College of Medicine and Penn State Health Milton S. Hershey Medical Center, Temple University, University of Michigan, University of Pittsburgh Medical Center, AdventHealth, Orlando Health System, University of Florida Health, University of Miami. These sites represent academic and community health systems. Patients who receive care in these institutions reside across all 50 states; Washington, DC; Puerto Rico; US Virgin Islands; and Guam.

b Age category based on patient age at the time of first encounter during the 1-year period.

c Other/missing not shown.

d Not shown: non-Hispanic American Indian or Alaska Native (<1%), non-Hispanic multiple race (<1%), non-Hispanic Native Hawaiian or Other Pacific Islander (<1%), other (2%), and missing (6%–9%; there were some variations by year).

e Body mass index (BMI) data were missing from 25% to 35% of patients. BMI category percentages have been calculated among those with available BMI data. BMI calculation: weight in kilograms divided by the square of height in meters.

### Preventive service use

**Figure 1:**
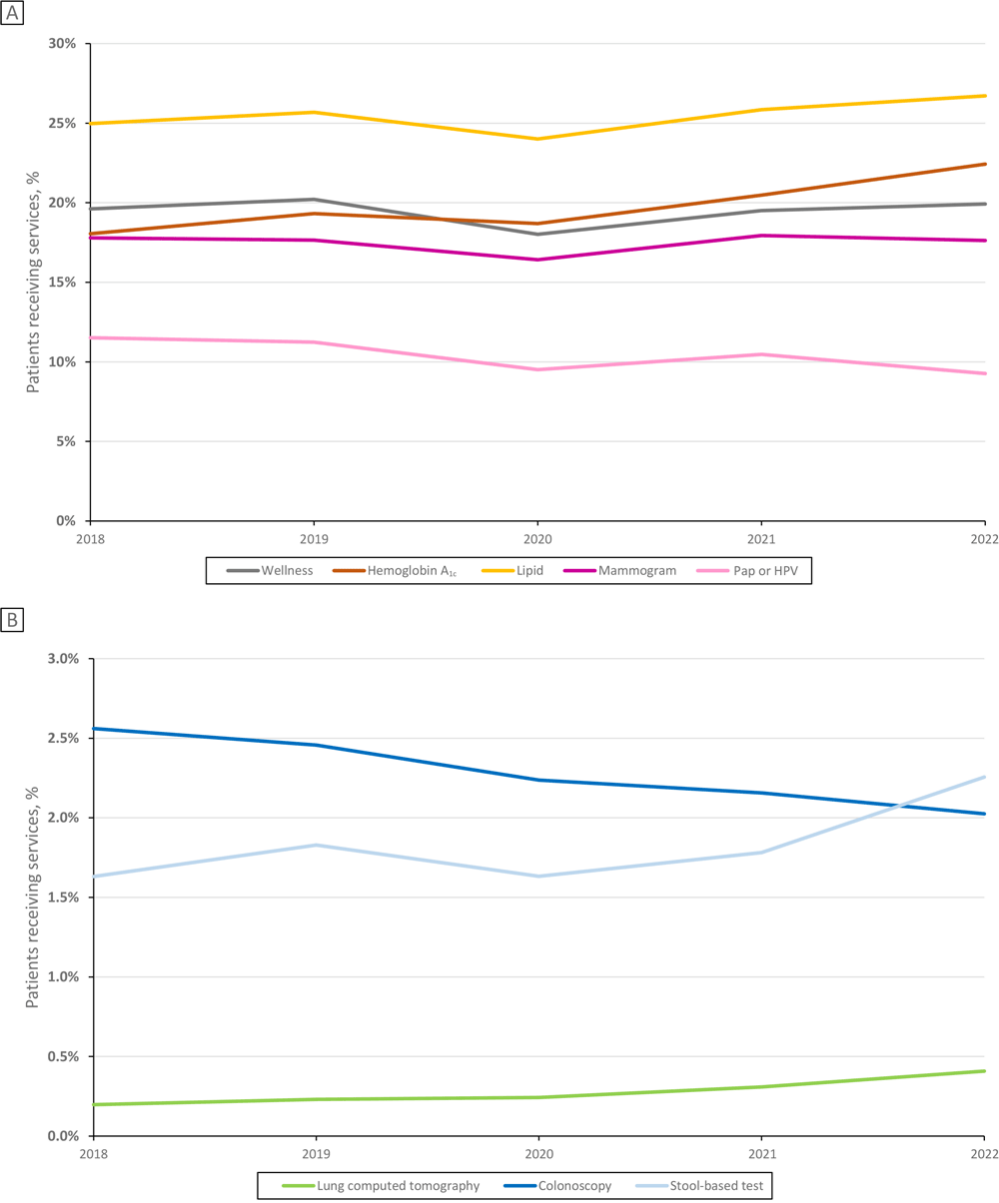
Preventive service usage among unique patients, National Patient-Centered Clinical Research Network (PCORnet) sites, 2018–2022. Percentages of patients receiving mammograms and Papanicolaou (Pap) tests or human papillomavirus (HPV) tests are presented out of all women receiving care that year from participating PCORnet sites; percentages of all other preventive services are presented out of all patients (men and women) receiving care that year from participating PCORnet sites. Colonoscopy indicates colonoscopy or sigmoidoscopy. Stool-based test indicates stool-based colorectal test. Total unique patient counts by year: 2018: N = 12,236,819 overall, 7,247,376 women; 2019: N = 13,026,666 overall, 7,695,294 women; 2020: N = 13,353,937 overall, 7,820,064 women; 2021: N = 15,590,610 overall, 9,127,882 women; 2022: N = 13,826,891 overall, 8,146,879 women.

During 2018 through 2022 (Figure 1), there was a significant increasing trend in usage of some preventive services, including HbA_1c_ testing (18% to 22%, *P* = .03) and lung CT scans (0.2% to 0.4%, *P* < .01). Usage of other preventive services did not change significantly: lipid testing, approximately 26%; wellness visits, 20%; mammograms among women, 18%; Pap tests or HPV tests among women, approximately 10%; and stool-based screening tests, 2%. However, there was a significant increasing trend in stool-based screening among those aged 45 to 49 years, with prevalence increasing from 0.6% in 2018 to 4.6% in 2022 (*P* = .02; shown in Supplemental Figure 1, available at https://stacks.cdc.gov/view/cdc/156743). Among all patients, there was a significant decreasing trend in colonoscopies or sigmoidoscopies, from approximately 3% to 2% (*P* < .01).

**Table:** 

Preventive service	2018, %	2019, %	2020, %	2021, %	2022, %
Wellness	19.6	20.2	18.0	19.5	19.9
Hemoglobin A_1c_	18.1	19.3	18.7	20.5	22.4
Lipid	25.0	25.7	24.0	25.8	26.7
Mammogram	17.8	17.7	16.4	17.9	17.6
Papanicolaou test or human papillomavirus test	11.5	11.2	9.5	10.5	9.3
Lung computed tomography	0.2	0.2	0.2	0.3	0.4
Colonoscopy	2.6	2.5	2.2	2.2	2.0
Stool-based test	1.6	1.8	1.6	1.8	2.3

Most preventive services experienced a decrease in usage during 2020 compared with 2019 (Figure 1). Examining the monthly distribution of usage of each preventive service during 2020, few preventive services occurred during April and May compared with the rest of the year (Supplemental Figure 2, available at https://stacks.cdc.gov/view/cdc/156743). There was estimated health debt for nearly all preventive services in 2020, although lung CT scans exceeded prepandemic expected levels (113%; Table 2). By 2022, all but 2 preventive services (Pap tests or HPV tests, 81%; colonoscopies or sigmoidoscopies, 81%; Table 2) had rebounded to, or exceeded, prepandemic levels (≥99%).

**Table 2:** Estimated Health Debt for Use of Preventive Services and Diagnoses of New Chronic Disease in 2020–2022 Compared With 2018–2019, National Patient-Centered Clinical Research Network (PCORnet)^a^

Service or diagnosis	2020	2021	2022	2020–2022
**Preventive services, %**				
Wellness visit	90	98	100	**96**
Hemoglobin A_1C_	100	110	120	**110**
Lipid testing	95	102	105	**101**
Colonoscopy	89	86	81	**85**
Stool-based test	94	103	130	**109**
Lung computed tomography	113	144	190	**149**
Mammogram	93	101	99	**98**
Papanicolaou tests or human papillomavirus tests	84	92	81	**86**
**New chronic disease diagnoses, %**				
Hypertension	90	98	99	**96**
Diabetes	87	92	100	**93**
Cholesterol	90	96	106	**98**
Colorectal cancer	90	91	97	**93**
Colonic adenoma	81	93	105	**94**
Lung cancer	89	85	88	**87**
Breast cancer	92	95	97	**95**
Cervical cancer	87	81	83	**84**
Cervical intraepithelial neoplasia or carcinoma in situ	90	87	87	**88**

a The average from 2018 to 2019 was used as the baseline for estimation of health debt. The observed annual numbers were then divided by the expected annual numbers, for each year individually (2020, 2021, and 2022), as well as collectively (2020–2022). Percentages indicate health debt if less than 100%. Health debt for mammograms, Papanicolaou tests and/or human papillomavirus tests, breast cancer, cervical cancer, and cervical intraepithelial neoplasia or carcinoma in situ are presented for all women; all other health debt estimates are for all patients (men and women). Colonoscopy indicates colonoscopy or sigmoidoscopy. Stool-based test indicates stool-based colorectal test. Cholesterol indicates initiation of a new cholesterol medication.

There was variation by race and ethnicity in preventive service usage (Supplemental Figure 3, available at https://stacks.cdc.gov/view/cdc/156743). For example, in 2022, non-Hispanic Asian adults had the highest percentage receiving lipid panel testing (34%) and wellness visits (26%); Hispanic adults had the highest percentage receiving HbA_1c_ tests (32%), Pap tests or HPV tests (15% of women), and stool-based screening tests (6%); and non-Hispanic White adults had the highest percentage receiving mammograms (20% of women) and lung CT scans (0.6%).

Preventive service trends showed some variation by race and ethnicity from 2018 to 2022. For example, there was a significant decreasing trend in colonoscopy or sigmoidoscopy among non-Hispanic White adults (*P* < .01; Supplemental Figure 4, available at https://stacks.cdc.gov/view/cdc/156743). All race and ethnicity groups had a significant increase in lung CT scans (*P* < .05 for all).

### New chronic disease diagnoses

**Figure 2:**
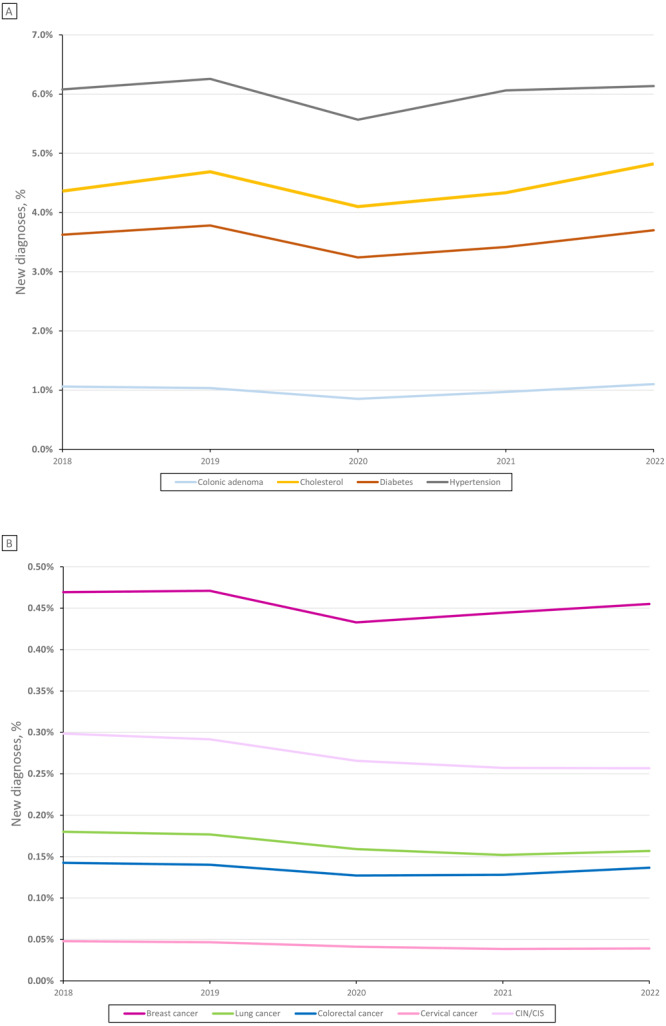
New chronic disease diagnoses among unique patients, National Patient-Centered Clinical Research Network (PCORnet) sites, 2018–2022. Percentages of patients newly diagnosed with breast cancer, cervical cancer, and cervical intraepithelial neoplasia or carcinoma in situ (CIN/CIS) are presented out of all women receiving care that year from participating PCORnet sites; percentages of patients diagnosed with all other conditions are presented out of all patients (men and women) receiving care that year from participating PCORnet sites. Cholesterol indicates initiation of a new cholesterol medication. Total unique patient counts by year: 2018: N = 12,262,754 overall, 7,264,953 women; 2019: N = 13,064,373 overall, 7,719,031 women; 2020: N = 13,402,584 overall, 7,849,336 women; 2021: N = 15,652,210 overall, 9,164,607 women; 2022: N = 15,506,288 overall, 9,128,281 women.

From 2018 to 2022, annual chronic disease diagnoses remained mostly stable, with a dip during 2020 (Figure 2). Over this period, approximately 6% of patients annually received a new hypertension diagnosis, 4% to 5% began a cholesterol medication, and 4% received a new diabetes diagnosis. Approximately 1% had a new colonic adenoma; 0.1% had new colorectal cancer; and among women, 0.5% had a new diagnosis of breast cancer. There was a significant decreasing trend in CIN/CIS among women (0.30% in 2018 to 0.26% in 2022, *P* = .01), cervical cancer among women (0.05% to 0.04%, *P* = .02), and lung cancer among all patients (0.18% to 0.16%, *P* = .04). For colonic adenoma, there was an increasing trend in new diagnoses among those aged 45 to 49 years (0.7% to 1.9%, *P* < .05; Supplemental Figure 1, available at https://stacks.cdc.gov/view/cdc/156743). Among patients aged 45 to 49 years, approximately 60 patients per 100,000 were diagnosed with colonic adenoma during 2018 through 2020; this increased to 88 per 100,000 in 2021 and 179 per 100,000 in 2022.

**Table:** 

Condition	2018, %	2019, %	2020, %	2021, %	2022, %
Colonic adenoma	1.06	1.03	0.85	0.97	1.10
Cholesterol	4.36	4.69	4.10	4.33	4.82
Diabetes	3.63	3.78	3.24	3.42	3.70
Hypertension	6.08	6.26	5.57	6.06	6.14
Breast cancer	0.47	0.47	0.43	0.44	0.46
Lung cancer	0.18	0.18	0.16	0.15	0.16
Colorectal cancer	0.14	0.14	0.13	0.13	0.14
Cervical cancer	0.05	0.05	0.04	0.04	0.04
CIN/CIS	0.30	0.29	0.27	0.26	0.26

The monthly distribution of new chronic disease diagnoses during 2020 showed few new diagnoses during April and May compared with the rest of the year (Supplemental Figure 5, available at https://stacks.cdc.gov/view/cdc/156743). There was estimated health debt for all chronic diseases in 2020, with observed levels ranging from 81% (colonic adenoma) to 92% (breast cancer) of expected prepandemic levels (Table 2). In 2022, new diagnoses of 3 conditions (hypertension, diabetes, and colonic adenoma) and new prescriptions for cholesterol medications had rebounded to, or exceeded, prepandemic levels (≥99%). However, any excess during 2022 was not sufficient to make up for the lost volume of diagnoses in 2020 and 2021. Over the combined 3-year pandemic period, 2020 through 2022, health debt remained for all 9 conditions. For the combined period 2020 through 2022, 3-year health debt ranged from 84% (cervical cancer) to 98% (new cholesterol medications).

Chronic disease diagnoses varied by race and ethnicity (Supplemental Figure 6, available at https://stacks.cdc.gov/view/cdc/156743). For example, in 2022, non-Hispanic Black adults had the highest percentage of new hypertension (8%) and diabetes (5%) diagnoses. Hispanic women had the highest percentage of new CIN/CIS (0.4%). During 2018 through 2022, diagnoses of new CIN/CIS decreased significantly among non-Hispanic White women (*P* < .01), and diagnoses of new cervical cancer decreased significantly among both non-Hispanic White (*P* < .01) and Hispanic (*P* = .02) women (Supplemental Figure 7, available at https://stacks.cdc.gov/view/cdc/156743).

## Discussion

In this EHR-based assessment of over 30 million US adults receiving care during 2018 through 2022, usage of preventive services and new diagnoses of chronic disease decreased during 2020, especially in the initial months of the COVID-19 pandemic. Preventive services and chronic disease diagnoses rebounded during 2021 and 2022, returning to nearly prepandemic levels, but health debt remained for several preventive services and chronic disease diagnoses. Use of EHR data allowed for stratification by sociodemographic factors, revealing racial and ethnic screening disparities as well as differential trends by age for those affected by colorectal cancer screening guideline changes (2). Overall, these findings highlight the usefulness of PCORnet data for chronic disease surveillance.

The observed decrease in preventive service usage during 2020 is consistent with prior work, although most existing studies were limited to specific settings, populations, or conditions. Researchers have reported decreases during 2020 for routine medical visits (4), HbA_1c_ testing (13), lipid testing (13,14), mammograms (7), colonoscopies (7), Pap tests (7), and lung CT scans (15,16). Telehealth and home-based services with clinical support, such as self-measured blood pressure monitoring (17,18), blood glucose monitoring (19,20), home-based HPV screening kits (21,22), and at-home colorectal screening (7) can reduce barriers to care and may have partially mitigated disruption from the pandemic, at least for some tests and diagnoses (14). The pandemic’s effects on screening choices may have contributed to results of this study, such as the observed decrease in colonoscopies or sigmoidoscopies and simultaneous increase in stool-based testing. Expanded implementation of alternative, more accessible modalities of care could support future chronic disease prevention, detection, and management.

Similar to the observed decrease in preventive service usage, there was a decrease in new chronic disease diagnoses during 2020. This finding was expected and is consistent with prior studies. For example, pandemic-related decreases occurred in cancer diagnoses (23–26), including colorectal, female breast, cervical, and lung cancer, as well as hypertension diagnostic procedures (27) and new statin prescriptions (13). Although diagnoses rebounded in 2021 and 2022, any delay in diagnosis can delay treatment, potentially leading to worse outcomes for affected patients. In a national, hospital-based registry capturing about 70% of all US cancer diagnoses annually, the proportion of cancers detected at early stages decreased during 2020 (24); also, a modeling study from England suggested that pandemic-related diagnostic delays for colorectal, breast, and lung cancers could have resulted in approximately 15%, 8%, and 5% increases, respectively, in deaths over 5 years (28). In addition, patients with chronic conditions such as diabetes, hypertension, and cancer are at increased risk of severe outcomes from COVID-19 (29,30), underscoring the importance of continued diagnosis and management of chronic conditions even amidst acute pandemic phases.

Racial and ethnic disparities are well documented for screening and burden of chronic disease. For example, non-Hispanic Black men have higher incidence of lung cancer than non-Hispanic White men; however non-Hispanic Black men are less likely to be screened, receive later-stage diagnoses, have lower rates of treatment, and have more deaths from lung cancer (31). In our study, non-Hispanic Black adults had lower receipt of lung CT scans compared with non-Hispanic White adults. In a prior study, non-Hispanic Black women had higher breast cancer mortality than non-Hispanic White women (32); however, another study found that non-Hispanic Black women were more likely to meet breast cancer screening guidelines than non-Hispanic White women (33). Our results were inconsistent with this prior study in that we observed lower mammography screening in non-Hispanic Black women compared with non-Hispanic White women. This difference may have been due to differences in population, measurement, or methodology. To some extent, our results stratified by race and ethnicity, with no further adjustment for age differences between these populations, may mask the degree of inequality. For example, a much younger age distribution among communities of color may partly explain the higher observed prevalence of Pap tests or HPV testing among Hispanic and non-Hispanic Asian women compared with non-Hispanic White women.

Age stratification revealed a sharp increase in both colonoscopy and stool-based screening among patients aged 45 to 49 in 2021 and 2022, corresponding to the 2021 USPSTF recommendation lowering the colorectal cancer screening threshold from age 50 to age 45 (2). Among this age group, we also observed increased diagnoses of new colonic adenoma. However, the observed volume of colorectal screening test usage remained low, consistent with prior estimates that nearly 30% of eligible US adults were not up to date with screening in 2021 (34). The ability to track implementation and potential impact of new screening recommendations or guidelines in clinical practice with timely, real-world data is a unique strength of EHR-based surveillance using PCORnet.

### Limitations

This study had several limitations. First, PCORnet is not a nationally representative sample of the US population: PCORnet has overrepresentation in academic health care settings and represents a care-seeking population (ie, patients who have some degree of access to and engagement in a health system). Second, included patients could have also obtained preventive services and diagnoses from other facilities that did not contribute data to PCORnet. Third, denominators for each preventive service were all adults, or all women, rather than the specific population recommended for each service based on age, risk factors, and recommended screening time intervals. Thus, this study was not designed to estimate whether patients were individually up to date with screening recommendations. Fourth, some patients may have received preventive care services as disease monitoring rather than screening; thus, our estimates likely overestimate the percentage of patients who received these tests for screening purposes alone. Furthermore, some lung CT scans during the COVID-19 pandemic may reflect pulmonary evaluations for COVID-19 rather than lung cancer screenings. Fifth, if a patient’s first encounter with a PCORnet health system occurred during our study period and led to the first documentation of a chronic disease, then some prevalent cases could have been included as new chronic disease diagnoses. Thus, our estimates for new diagnoses may be higher than actual incidence. Sixth, it is impossible to disentangle whether any changes in recorded new diagnoses were a consequence of altered testing and treatment during the pandemic rather than other secular trends that may have occurred even without the pandemic, such as increasing disease incidence or declining health care usage. Seventh, some patients could have been represented in the data more than once if they received care from multiple PCORnet sites. Finally, as EHR data are real-world data, they are subject to limitations such as missingness and variation across sites; further analyses could replicate these findings using varied data sources and phenotype definitions.

Despite these limitations, trends show a clear national level health debt with a drop in usage of preventive services and new chronic disease diagnoses during 2020 through 2022, despite some services rebounding to prepandemic levels. If trends in preventive services or diagnoses were not flat during the prepandemic period (2018–2019), then our method of estimating health debt could lead to overestimates (if trends were declining) or underestimates (if trends were increasing). Also, if fewer patients received care of any kind during the pandemic period (2020–2022), our method may underestimate health debt, since expected numbers of services and diagnoses were based on the number of patients who had encounters in institutions participating in PCORnet. Furthermore, while our study examined trends in yearly cross-sectional data, a longitudinal study examining patient-level data could better assess whether results are clinically meaningful, and what opportunities might exist for public health intervention. Future work could investigate health debt in more detail, including geographic variation, underlying causes of trends (such as health care capacity or delivery issues, or misinformation among patients), factors associated with returning to care, populations most affected by health debt, and potential effects of delayed diagnoses, later-stage disease detection, or delayed treatment initiation. Further exploration of incidence and services, such as whether eligible patients are up to date with their screenings, will be important for informing public health activities. In support of future efforts, this study’s codes and methodology have been shared publicly for replication, refinement, and use.

### Conclusions

This multisite, nationwide assessment of over 30 million patients revealed a decrease in both preventive services and new chronic disease diagnoses during the first year of the COVID-19 pandemic, followed by an increase in 2021 and 2022 to nearly prepandemic levels. In addition to examining health debt in preventive services and chronic disease diagnoses during the COVID-19 pandemic, PCORnet data also allow for examination of disparities by race and ethnicity as well as implementation of new screening recommendations. Overall, this study highlights ways to augment traditional chronic disease surveillance with data from PCORnet or other EHR-based surveillance systems — for timely assessment of emerging trends in chronic disease nationwide.
